# Retrospective Case‐Cohort Study on Risk Factors for Developing Distant Metastases in Women With Breast Cancer

**DOI:** 10.1002/cam4.70903

**Published:** 2025-04-18

**Authors:** Serena Bertozzi, Ambrogio Pietro Londero, Giovanni Vendramelli, Maria Orsaria, Laura Mariuzzi, Enrico Pegolo, Carla Di Loreto, Carla Cedolini, Vincenzo Della Mea

**Affiliations:** ^1^ Breast Unit University Hospital of Udine, ASUFC Udine Italy; ^2^ Department of Neuroscience, Rehabilitation, Ophthalmology, Genetics, Maternal and Infant Health University of Genoa Genova Italy; ^3^ Obstetrics and Gynecology Unit IRCCS Istituto Giannina Gaslini Genova Italy; ^4^ Institute of Pathology University Hospital of Udine Udine Italy; ^5^ Department of Mathematics, Computer Science and Physics University of Udine Udine Italy

**Keywords:** breast cancer metastasis, distant recurrence prediction, machine learning, personalized treatment, progesterone receptor, risk factors, tumor subtype

## Abstract

**Objective:**

This study aimed to identify risk factors associated with the development of metastases in breast cancer patients, to investigate survival rates, and the relationship between local recurrences and distant metastases.

**Methods:**

This retrospective case‐cohort study included women with breast cancer who were treated at a certified Breast Unit between 2001 and 2015. Cases who developed distant metastases were compared to controls based on diagnosis year, stage, and age at diagnosis. Comprehensive information on patient characteristics, tumor biology, and treatment options was gathered.

**Results:**

The study included 412 patients who developed distant metastases and 433 controls who remained metastasis‐free over a median follow‐up of 150 months (interquartile range 87–202). The 20‐year overall survival was 99.23% for the control group and 23.62% for those with metastasis (*p* < 0.01). Significant risk factors for metastasis included lobular invasive carcinoma (odds ratio (OR) 2.26, *p* < 0.001), triple‐negative subtype (OR 4.06, *p* = 0.002), high tumor grade (OR 2.62, *p* = 0.004), larger tumor size (OR 1.02, *p* < 0.001), lymph node involvement (*p* < 0.001), and loco‐regional recurrence (OR 4.32, *p* < 0.001). Progesterone receptor (PR) expression was protective (OR 0.52, 95% confidence interval 0.34–0.81, *p* = 0.003). Machine learning models supported these findings, though their clinical significance was limited.

**Conclusions:**

Lobular invasive carcinoma, specific tumor subtypes, high grade, large tumor size, lymph node involvement, and loco‐regional recurrence are all significant risk factors for distant metastasis, whereas PR expression is protective. The potential of machine learning in predicting metastasis was explored, showing promise for future personalized risk assessment.

**Abbreviations**
95% CI: 95% Confidence Interval
AJCC: American Joint Committee on Cancer
AUROC: Area Under the Receiver Operating Characteristic Curve
BCS: Breast‐Conserving Surgery
BMI: Body Mass Index
CALND: Complete Axillary Lymph Node Dissection
ER: Estrogen Receptor
FISH: Fluorescence In Situ Hybridization
HER2: Human Epidermal Growth Factor Receptor 2
IQR: Interquartile Range
MTM: Modified Total Mastectomy
NSM: Nipple‐Sparing Mastectomy
OR: Odds Ratio
PR: Progesterone Receptor
ROLL: Radio‐Guided Occult Lesion Localization
SD: Standard Deviation
SLNB: Sentinel Lymph Node Biopsy
SSM: Skin‐Sparing Mastectomy
SVM: Support Vector Machine
TNM: Tumor Node Metastasis
UICC: Union for International Cancer Control
VUS: Variant of Uncertain Significance

## 1 Introduction

Metastases in breast cancer are a critical factor influencing morbidity and mortality, with a significant percentage of deaths attributed to metastatic spread [1]. Despite advancements in breast cancer treatment, significant knowledge gaps remain, particularly regarding the factors that influence metastases development. The metastatic potential varies among different breast cancer subtypes, with triple‐negative breast cancer being associated with a shorter time to distant relapse and death [2, 3, 4]. However, the aggressive nature of triple‐negative breast cancer, which frequently leads to distant metastases through the hematogenous route rather than the lymphatic one, is not fully accounted for by traditional prognostic tools [4]. Therefore, understanding the risk factors and patterns of metastatic spread in breast cancer is crucial for prognosis prediction and treatment planning [5].

Bone is the most common site for breast cancer metastasis, with approximately 90% of deaths during treatment attributed to metastatic spread [6]. However, breast cancer metastases can manifest in various organs, including the brain, liver, lung, bone, and soft tissues, and they are associated with poor prognosis and decreased overall survival [3, 4]. Different breast cancer subtypes exhibit varying patterns of metastatic spread, with unique gene signatures potentially influencing the organs targeted by metastatic cells [7]. Factors such as estrogen receptor status and carcinoma subtype play a role in determining the site of metastasis [8].

While targeted management strategies may improve outcomes by addressing specific risk factors [3], research shows that local recurrence significantly increases the risk of distant metastasis [9, 10, 11, 12, 13]. This connection is further evidenced by the fact that breast cancer cutaneous metastases are frequently located near the primary tumor, suggesting a role for local lymphatic drainage [14]. As a consequence, effective local control with appropriate surgical margins or adjuvant radiotherapy after surgery and targeted prevention strategies are crucial for reducing distant metastasis and improving overall survival [9, 15].

The primary objective of the present study was to evaluate the risk factors for the development of metastatic sites in patients with breast cancer. The secondary objectives were to assess survival and the association between local recurrences and the development of distant metastases.

## 2 Methods

### 2.1 Study Design, Setting, and Sample

We conducted a retrospective case‐cohort study to examine factors associated with distant metastases in breast cancer patients. To identify the subcohort, the entire cohort of women with breast cancer treated at the Breast Unit between 2001 and 2015 was considered. The primary variable of interest was the development of distant metastases during follow‐up. A case‐cohort sampling design was used to ensure a balanced comparison while controlling for confounding variables. This design enabled the efficient extraction of cases and a subcohort of non‐cases that correspond to them. Each case was matched with a control using the following matching variables: year of diagnosis, stage, and age at diagnosis. The study was approved by the local Internal Review Board and followed the Helsinki Declaration. Data processing followed the dictates of the general authorization of the Italian Data Protection Authority to process personal data for scientific research purposes.

### 2.2 Measurement and Data Collection

The study included cases of invasive breast carcinomas treated during the specified period, who did not present metastases at the time of diagnosis. All included cases had complete information available on follow‐up data for at least 12 months after the oncological breast surgery.

Data for patient selection and data analysis were gathered from the clinical files of outpatient clinics, breast surgery, operative theater registers, and the pathology information system. We collected comprehensive data about patient, tumor, and treatment to understand the factors associated with the development of breast cancer distant metastases. Patient characteristics included age, recorded in years and categorized into age groups: < 45, 45–69, and ≥ 70 years [16, 17]. Menopausal status was categorized as pre‐menopausal or post‐menopausal. We documented the eventual family history for the presence of breast or ovarian cancer in family members. We collected genetic testing results, categorizing them as negative, variants of uncertain significance (VUS), or positive for known pathogenetic mutations. We also recorded body mass index (BMI) (kg/m^2^), tobacco smoke habits, and the use of estro‐progestinic therapies. To account for changes in treatment modalities over time, the overall treatment period was partitioned into two equal‐duration intervals. Each patient was then assigned to the interval during which the majority (i.e., more than 50%) of her treatments were administered.

Considered tumor characteristics included histology types such as invasive carcinoma of non‐special type, lobular invasive carcinoma, and ductal and lobular invasive carcinoma. We also classified breast cancer molecular subtypes as follows: luminal A, luminal B HER2‐, luminal B HER2+, HER2‐enriched, and triple‐negative [18, 19]. Tumor diameter was measured in millimeters, and we assessed the presence or absence of lymphatic invasion and perineural invasion. Hormone receptor status was evaluated, with estrogen receptor (ER) and progesterone receptor (PR) expressions categorized as negative or positive (≥ 1% in any nuclear staining) [20], including percentage positivity. HER2/Neu expression was measured using immunohistochemistry and classified as negative, 1+, 2+, or 3+. HER2/Neu FISH amplification was also performed, and HER2/Neu was defined as overexpressed/amplified when staining 3+ or 2+ with FISH amplification and as negative if the value was 0, 1+, or 2+ without FISH amplification [20]. Mib1/Ki‐67 positivity was measured as a percentage and categorized into low (< 20%) and high (> 20%).

Lymph node characteristics were also documented, including the total number of sentinel lymph nodes, sentinel lymph node status (non‐metastatic, micrometastasis, or macrometastasis), and the total number of metastatic lymph nodes. Additionally, we recorded the total number of lymph nodes extracted, the total number of lymph nodes with isolated tumoral cells, micrometastasis, and macrometastasis.

The number of local recurrences was categorized as no recurrences, one recurrence, or more than one recurrence. Tumor staging was detailed, recording local tumor extension (T1, T2, T3, T4), nodal status (N0, N1, N2, N3), TNM stage (I, II, III, IV), and nuclear grading (G1, G2, G3).

Pathological specimens were routinely examined following European guidelines [21, 22]. Samples smaller than 30 mm were completely sliced and evaluated, whereas specimens larger than 30 mm were sampled in accordance with European guidelines [21, 22]. The histology and nodal status were determined using World Health Organization criteria [23] and TNM classification VII (AJCC/UICC, 2009) [24]. The recommendations made by Elston Ellis were taken into account during the grading process [25].

### 2.3 Treatment Options Including Surgical and Non‐Surgical Approaches

Surgical oncology management documentation included the specific type of breast surgery performed, which could be either breast‐conserving surgery (BCS) or mastectomy. The type of axillary surgery was also noted, which could be sentinel lymph node biopsy (SLNB) or complete axillary lymph node dissection (CALND). Non‐surgical treatment variables included administering adjuvant hormonal therapy, adjuvant or neoadjuvant chemotherapy, adjuvant anti‐Her2 therapy, and adjuvant radiation therapy.

The surgical techniques described in this study were based on previously established procedures for BCS and mastectomy [11, 26]. BCS entailed the excision of the cancerous growth, followed by intra‐operative radiologic and/or pathologic specimen examination to confirm macroscopic margin negativity. Surgeons used wire‐hook placement or radio‐guided occult lesion localization (ROLL) for non‐palpable lesion localization. Mastectomy techniques reported in this study were modified total mastectomy (MTM), skin‐sparing mastectomy (SSM), and nipple‐sparing mastectomy (NSM). The choice of technique was determined by clinical indications and the patient's preference [11]. Axillary surgery included sentinel lymph node biopsy (SLNB) technique and/or complete axillary lymph node dissection (CALND) when appropriate. The SLNB procedure consisted of the pre‐operative intradermal injection of 99 m‐technetium radiolabeled tracer and its intra‐operative detection through a gamma probe [27, 28].

Radiation therapy after BCS was advised unless there were contraindications based on age or other medical conditions. Patients diagnosed with tumors that have spread to nearby tissues received radiotherapy to the chest wall and lymph node stations as deemed necessary. The decision to administer adjuvant chemotherapy and/or endocrine therapy was stated through multidisciplinary team discussion, taking into account tumor and patient characteristics, as well as the patient's preference.

### 2.4 Data Analysis and Machine Learning

A first preliminary machine learning experiment has been conducted to evaluate whether there was some probability of predicting distant relapse from the sole data available for each case, excluding age (because it was used to select the cohort). For this analysis, the patient cohort was divided into a training set (80%) and a validation set (20%) to develop and test the models. Five‐fold cross‐validation experiments have been carried out with different algorithms using Weka 3.8.6 (University of Waikato, Hamilton, New Zealand). The algorithms involved in the investigation are NaiveBayes, SVM, Logistic, Multilayer Perceptron, K*, AdaBoost, Bagging, and RandomForest.

Other statistical analyses were performed using R (version 4.4.0—http://www.r‐project.org/). The normal distribution of the numerical variables was assessed using the Kolmogorov–Smirnov test. Numerical variables were described with mean and standard deviation (SD) if they followed a parametric distribution or median and interquartile range (IQR) if they followed a non‐parametric distribution. Categorical variables were described as percentages and absolute values. A weighted Cox model was used to analyze overall survival (OS). In OS analysis, only deaths from cancer were considered. A weighted logistic regression was used to analyze the factors related to the occurrence of distant metastases (dependent factor) and the results are reported as odds ratios (OR) with 95% confidence intervals (95% CI). Random imputation was used to assess the influence of missing data on the logistic regression models. Spearman's test was used for correlation analyses.

## 3 Results

**FIGURE 1:**
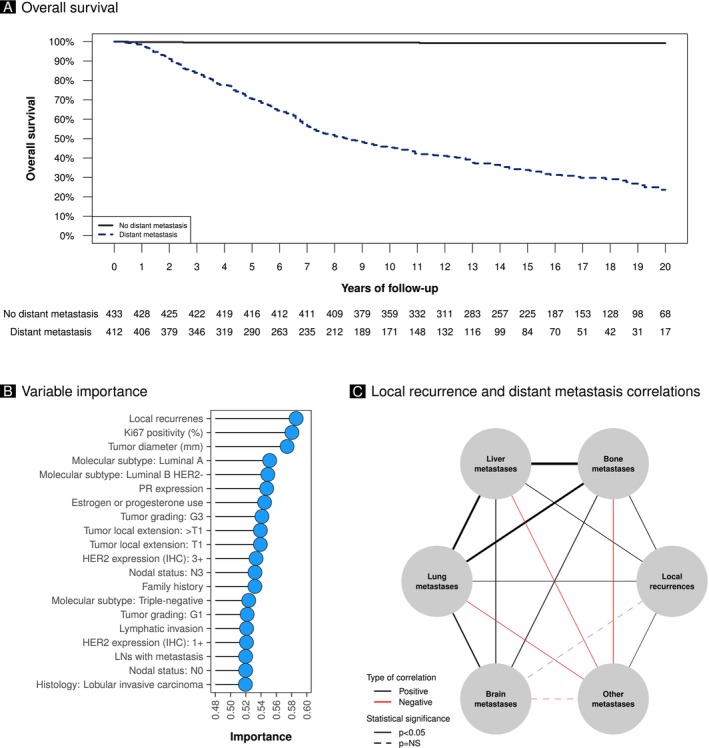
(A) Survival curves between the group that developed distant metastases and the group that did not. In this analysis, only deaths from cancer were considered. Weighted Cox model, *p* < 0.05. (B) This plot shows the variable importance estimated with a Random Forest model. (C) Network of correlations between loco‐regional recurrences and the different types of distant metastases.

The study included 412 patients with breast cancer who developed metastases during their follow‐up and 433 controls who remained metastasis‐free. The median follow‐up length was 150 months (IQR 87–202). The overall survival was 99.23% (95% CI 98.37%–100%) at 20 years follow‐up in the control group and 23.62% (95% CI 18.48%–30.19%) in the group that developed distant metastases (*p* < 0.01) (Figure 1A). Table 1 shows patients' age and tumor stage in cases, controls, and the original cohort. The median age of the study population was 57 years (IQR 47–67). Approximately 20% of patients reported a family history of breast or ovarian cancer (Table 1).

**TABLE 1:** Matching variables in the case‐cohort sampling: distribution of age and TNM stage across cases, control group, and the full cohort.

	No distant metastasis (433)	Distant metastasis (412)	Full cohort (3861)
Woman age (years)	57.00 (47.00–67.00)	57.00 (46.00–67.00)	61.00 (50.00–69.00)
Age categories			
< 45 years	19.86% (86/433)	19.17% (79/412)	11.73% (453/3861)
45–69 years	60.97% (264/433)	61.41% (253/412)	65.24% (2519/3861)
≥ 70 years	19.17% (83/433)	19.42% (80/412)	23.03% (889/3861)
TNM stage			
I	26.56% (115/433)	26.46% (109/412)	56.77% (2192/3861)
II	35.80% (155/433)	34.47% (142/412)	28.8% (1112/3861)
III	37.64% (163/433)	39.08% (161/412)	14.43% (557/3861)

Tables 2, 3 and 4 analyze the differences between the group that developed metastases during follow‐up and those that did not. Among the 60 patients who underwent genetic testing, 27% were found to have a pathogenic mutation, while 15% had a variant of uncertain significance. The non‐special type histotype (formerly known as invasive ductal carcinoma) accounted for the majority of cases (75%) (Table 2). Among molecular subtypes, luminal subtypes were predominant (approximately 80%), with a smaller proportion of triple‐negative tumors (approximately 12%) or pure HER2‐positive tumors (approximately 8%) (Table 3). The median Mib1/Ki‐67 was found to be 20%, which also served as the cutoff in our study population (Table 3). About 13% of the study population experienced a loco‐regional recurrence (Table 3). The initial stage at diagnosis was stage I in about 27% of patients, stage II in 35%, and stage III in 38%. The tumor grade was G1 in 8% of patients, G2 in 55%, and G3 in 37% (Table 4).

**TABLE 2:** Comparison of population characteristics between patients with and without distant metastases.

Patient characteristics	No distant metastasis (433)	Distant metastasis (412)	OR (95% CI)	*p*
BMI (kg/m^2^)	25.00 (22.00–28.00)	24.00 (22.00–28.00)	1.01 (0.99–1.03)	0.481
Family history	28.00% (49/175)	15.92% (57/358)	0.58 (0.43–0.79)	< 0.001
Genetic testing				
Genetic testing negative	58.82% (10/17)	58.14% (25/43)	Reference	1.000
Any VUS	0.00% (0/17)	20.93% (9/43)	—	—
Any positivity for genetic predisposition	41.18% (7/17)	20.93% (9/43)	1.11 (0.44–2.78)	0.828
Tobacco smoke	7.91% (28/354)	7.95% (29/365)	0.78 (0.52–1.16)	0.213
Estrogen or progesterone use	36.05% (31/86)	25.71% (18/70)	0.92 (0.53–1.62)	0.779
Menopausal status				
Pre‐menopausal	32.41% (140/432)	29.93% (123/411)	Reference	1.000
Post‐menopausal	67.59% (292/432)	70.07% (288/411)	0.66 (0.53–0.83)	< 0.001
Surgical treatment				
Breast surgery				
Mastectomy	54.37% (230/423)	64.39% (255/396)	Reference	1.000
BCS	45.63% (193/423)	35.61% (141/396)	0.40 (0.32–0.49)	< 0.001
Axilla surgery				
CALND	73.67% (319/433)	79.13% (326/412)	Reference	1.000
SLNB	26.33% (114/433)	20.87% (86/412)	0.25 (0.2–0.32)	< 0.001
Non‐surgical treatment				
Hormonal therapy	77.37% (335/433)	76.46% (315/412)	0.70 (0.55–0.89)	0.004
Adjuvant chemotherapy	53.81% (233/433)	72.57% (299/412)	5.42 (4.32–6.81)	< 0.001
Trastuzumab	11.78% (51/433)	8.98% (37/412)	1.45 (1.01–2.09)	0.045
Radiotherapy	60.51% (262/433)	59.47% (245/412)	1.02 (0.83–1.26)	0.858

*Note:* This table presents the mean (±SD) or median (IQR) for continuous variables and percentages with absolute values for categorical variables. The univariate weighted logistic regression analysis results, including odds ratios (OR) with 95% confidence intervals (95% CI) and *p*‐values, are also displayed, with distant metastasis as the dependent variable.

**TABLE 3:** Comparison of tumor characteristics between patients with and without distant metastases.

Tumor characteristics	No distant metastasis (433)	Distant metastasis (412)	OR (95% CI)	*p*
Tumor histology				
Invasive carcinoma non‐special type	77.14% (334/433)	74.27% (306/412)	Reference	1.000
Lobular invasive carcinoma	14.55% (63/433)	18.45% (76/412)	1.75 (1.33–2.29)	< 0.001
Ductal and lobular invasive carcinoma	8.31% (36/433)	7.28% (30/412)	1.5 (1–2.25)	0.047
Breast cancer molecular subtype				
Luminal A	51.14% (180/352)	36.65% (129/352)	Reference	1.000
Luminal B HER2—	19.32% (68/352)	29.83% (105/352)	2.82 (2.14–3.72)	< 0.001
Luminal B HER2+	11.08% (39/352)	12.22% (43/352)	3.33 (2.28–4.86)	< 0.001
HER2+	8.81% (31/352)	6.53% (23/352)	3.73 (2.28–6.11)	< 0.001
Triple‐negative	9.66% (34/352)	14.77% (52/352)	3.12 (2.2–4.42)	< 0.001
Tumor diameter (mm)	19.50 (12.00–25.00)	21.50 (15.00–30.00)	1.04 (1.03–1.05)	< 0.001
Lymphatic invasion	20.79% (90/433)	25.00% (103/412)	2.01 (1.58–2.56)	< 0.001
Perineural invasion	0.23% (1/433)	0.49% (2/412)	5.12 (0.87–30.06)	0.07
Estrogen receptor (ER) Expression	82.48% (353/428)	80.20% (328/409)	0.51 (0.39–0.66)	< 0.001
Percentage of positivity of ER	90.00 (70.00–90.00)	90.00 (70.00–90.00)	0.99 (0.99–1)	< 0.001
Progesteron receptors (PR) expression	70.79% (303/428)	62.10% (254/409)	0.51 (0.41–0.64)	< 0.001
Percentage of positivity of PR	70.00 (40.00–90.00)	70.00 (40.00–90.00)	0.99 (0.99–1)	0.002
Overexpressed/amplified HER2	18.81% (73/388)	17.81% (70/393)	1.98 (1.49–2.64)	< 0.001
HER2 expression (IHC)				
Negative	36.13% (138/382)	35.20% (138/392)	Reference	1.000
1+	33.77% (129/382)	35.71% (140/392)	1.44 (1.13–1.85)	0.004
2+	13.87% (53/382)	14.03% (55/392)	1.48 (1.06–2.06)	0.022
3+	16.23% (62/382)	15.05% (59/392)	2.32 (1.66–3.24)	< 0.001
Ki67/Mib1 positivity (%)	20.00 (8.00–40.00)	27.50 (12.00–50.00)	1.02 (1.02–1.03)	< 0.001
Ki67/Mib1 positivity				
Low < 20%	57.60% (197/342)	43.45% (146/336)	Reference	1.000
High > 20%	42.40% (145/342)	56.55% (190/336)	2.86 (2.27–3.6)	< 0.001
Lymph node characteristics				
Total number of sentinel lymph nodes (sLN)	1.00 (1.00–2.00)	1.00 (1.00–2.00)	0.84 (0.66–1.07)	0.159
Sentinel lymph nodes status				
Non‐metastatic	54.40% (105/193)	58.00% (87/150)	Reference	1.000
Micrometastasis	9.84% (19/193)	12.67% (19/150)	1.38 (0.83–2.32)	0.215
Macrometastasis	35.75% (69/193)	29.33% (44/150)	1.99 (1.36–2.9)	< 0.001
LNs with metastasis	57.32% (235/410)	61.27% (242/395)	3.27 (2.64–4.06)	< 0.001
Total number of LNs extracted	17.00 (10.00–23.00)	17.00 (10.00–23.00)	1.06 (1.05–1.07)	< 0.001
Total number of LNs with metastasis	1.00 (0.00–4.00)	1.00 (0.00–6.00)	1.11 (1.09–1.14)	< 0.001
Total number of LNs with isolated tumoral cells	0.00 (0.00–0.00)	0.00 (0.00–0.00)	2.17 (1.04–4.56)	0.04
Total number of LNs with micrometastasis	0.00 (0.00–0.00)	0.00 (0.00–0.00)	1.12 (0.71–1.74)	0.628
Total number of LNs with macrometastasis	1.00 (0.00–4.00)	1.00 (0.00–6.00)	1.11 (1.09–1.13)	< 0.001
Outcomes				
Number of local recurrenes				
No recurrences	95.84% (415/433)	78.64% (324/412)	Reference	1.000
One recurrence	3.46% (15/433)	15.29% (63/412)	5.51 (3.97–7.64)	< 0.001
More recurrences	0.69% (3/433)	6.07% (25/412)	32.89 (14.49–74.65)	< 0.001

*Note:* This table presents the mean (±SD) or median (IQR) for continuous variables and percentages with absolute values for categorical variables. Results from the univariate weighted logistic regression analysis, including odds ratios (OR) with 95% confidence intervals (95% CI) and *p*‐values, are also displayed, with distant metastasis as the dependent variable.

**TABLE 4:** Comparison of tumor staging between patients with and without distant metastases.

Tumor staging	No distant metastasis (433)	Distant metastasis (412)	OR (95% CI)	*p*
Tumor local extension				
T1	56.12% (243/433)	48.30% (199/412)	Reference	1.000
T2	35.10% (152/433)	39.81% (164/412)	3.18 (2.54–3.97)	< 0.001
T3	5.08% (22/433)	7.04% (29/412)	6.76 (4.22–10.83)	< 0.001
T4	3.70% (16/433)	4.85% (20/412)	6.99 (3.99–12.27)	< 0.001
Nodal status				
N0	41.49% (178/429)	37.50% (153/408)	Reference	1.000
N1	28.90% (124/429)	27.45% (112/408)	2.02 (1.56–2.61)	< 0.001
N2	18.18% (78/429)	17.40% (71/408)	5.78 (4.19–7.96)	< 0.001
N3	11.42% (49/429)	17.65% (72/408)	8.57 (6.13–11.98)	< 0.001
Tumor grading				
G1	9.88% (42/425)	5.35% (22/411)	Reference	1.000
G2	57.41% (244/425)	53.28% (219/411)	3.35 (2.14–5.25)	< 0.001
G3	32.71% (139/425)	41.36% (170/411)	5.79 (3.67–9.14)	< 0.001

*Note:* This table presents percentages with absolute values for the categorical variables. Results from the univariate weighted logistic regression analysis, including odds ratios (OR) with 95% confidence intervals (95% CI) and *p*‐values, are also displayed, with distant metastasis as the dependent variable.

Patients who developed distant metastases during follow‐up were more likely to have undergone radical interventions both at the breast level (mastectomy) and at the axillary level (CALND), as well as adjuvant chemotherapy (Table 2). Indeed, cases showed statistically larger tumor sizes at diagnosis compared to controls, as well as a higher prevalence of triple‐negative and Luminal B molecular subtypes, and a lower prevalence of Luminal A subtype (Table 3). There was also a higher prevalence of Mib1/Ki‐67 > 20% in cases compared to controls, as well as G3 grading, possibly reflecting higher cell proliferation and, thus, greater intrinsic tumor aggressiveness among these patients (Tables 3 and 4). Cases also showed a higher prevalence of pN3 stages at diagnosis, indicating massive lymph node involvement at the time of diagnosis (Table 4).

Table 5 presents the analysis using univariate and multivariate weighted logistic regression on the potential predictive factors for the development of distant metastases in women with breast cancer. Lobular invasive carcinoma (OR 2.26, 95% CI 1.45–3.51, *p* < 0.001), triple‐negative molecular subtype (OR 4.06, 95% CI 1.70–9.72, *p* = 0.002), high tumor grade (> G1) (OR 2.62 95% CI 1.35–5.10, *p* = 0.004), larger tumor size (OR 1.02, 95% CI 1.01–1.04, *p* < 0.001), lymph node involvement at diagnosis (e.g., N2 OR 4.93, 95% CI 2.93–8.28, *p* < 0.001) and occurrence of loco‐regional recurrence (OR 4.32, 95% CI 2.76–6.74, *p* < 0.001) were significant risk factors for the development of distant metastases during the follow up (Table 5A). Meanwhile, the expression of progesterone receptors (PR) was a significant protective factor (OR 0.52, 95% CI 0.34–0.81, *p* = 0.003) (Table 5A). In Table 5B, a sensitivity analysis randomly imputing the missing values shows a similar pattern of findings. Moreover, accounting for the years of treatment, which serves as a proxy for treatment changes over time, did not alter the pattern of findings (Table 5).

**TABLE 5:** Predictive factors for the development of distant metastases.

(A) Distant metastases				
	OR (95% CI)	*p*	OR (95% CI)^a^	*p* ^a^
Family history	0.58 (0.43–0.79)	< 0.001	0.74 (0.49–1.14)	0.170
Tumor histology				
Invasive carcinoma non‐special type				
Lobular invasive carcinoma	1.75 (1.33–2.29)	< 0.001	2.26 (1.45–3.51)	< 0.001
Ductal and lobular invasive carcinoma	1.5 (1–2.25)	0.047	0.81 (0.42–1.59)	0.547
Breast cancer molecular subtype				
Luminal A				
Luminal B HER2—	2.82 (2.14–3.72)	< 0.001	2.45 (1.47–4.07)	< 0.001
Luminal B HER2+	3.33 (2.28–4.86)	< 0.001	2.00 (1.08–3.71)	0.027
HER2+	3.73 (2.28–6.11)	< 0.001	2.38 (0.92–6.17)	0.074
Triple‐negative	3.12 (2.2–4.42)	< 0.001	4.06 (1.70–9.72)	0.002
Tumor diameter (mm)	1.04 (1.03–1.05)	< 0.001	1.02 (1.01–1.04)	< 0.001
Nodal status				
N0				
N1	2.02 (1.56–2.61)	< 0.001	2.15 (1.49–3.11)	< 0.001
N2	5.78 (4.19–7.96)	< 0.001	4.93 (2.93–8.28)	< 0.001
N3	8.57 (6.13–11.98)	< 0.001	7.54 (4.18–13.62)	< 0.001
Progesterone receptors (PR) expression	0.51 (0.41–0.64)	< 0.001	0.52 (0.34–0.81)	0.003
Ki67/Mib1 positivity (%)	1.02 (1.02–1.03)	< 0.001	1.00 (0.98–1.01)	0.417
Tumor grading				
G1				
> G1	4.11 (2.65–6.37)	< 0.001	2.62 (1.35–5.10)	0.004
Local recurrences	7.21 (5.36–9.71)	< 0.001	4.32 (2.76–6.74)	< 0.001
Years of treatment				
2001–2007				
≥ 2008	0.54 (0.44–0.66)	< 0.001	0.33 (0.24–0.46)	< 0.001

(B) Distant metastases (impuration of missing values)				
	OR (95% CI)	p	OR (95% CI)^a^	*p* ^a^
Family history	0.47 (0.35–0.61)	< 0.001	0.71 (0.52–0.95)	0.023
Tumor histology				
Invasive carcinoma non‐special type				
Lobular invasive carcinoma	1.75 (1.33–2.29)	< 0.001	1.55 (1.12–2.13)	0.008
Ductal and lobular invasive carcinoma	1.5 (1–2.25)	0.047	0.97 (0.61–1.55)	0.898
Breast cancer molecular subtype				
Luminal A				
Luminal B HER2—	2.03 (1.58–2.62)	< 0.001	1.50 (1.04–2.16)	0.028
Luminal B HER2+	3.57 (2.53–5.03)	< 0.001	1.63 (1.05–2.53)	0.030
HER2+	3.14 (1.98–4.96)	< 0.001	1.01 (0.54–1.88)	0.976
Triple‐negative	2.91 (2.1–4.04)	< 0.001	1.49 (0.86–2.59)	0.152
Tumor diameter (mm)	1.04 (1.03–1.05)	< 0.001	1.02 (1.01–1.03)	< 0.001
Nodal status				
N0				
N1	1.96 (1.52–2.54)	< 0.001	1.90 (1.43–2.51)	< 0.001
N2	5.91 (4.3–8.13)	< 0.001	3.70 (2.6–5.27)	< 0.001
N3	8.77 (6.28–12.24)	< 0.001	5.80 (3.96–8.49)	< 0.001
Progesteron receptors (PR) expression	0.5 (0.41–0.62)	< 0.001	0.75 (0.55–1.01)	0.060
Ki67/Mib1 positivity (%)	1.02 (1.02–1.02)	< 0.001	1.00 (1.00–1.01)	0.338
Tumor grading				
G1				
> G1	4.22 (2.72–6.54)	< 0.001	2.35 (1.46–3.78)	< 0.001
Local recurrences	7.21 (5.36–9.71)	< 0.001	6.52 (4.65–9.14)	< 0.001
Years of treatment				
2001–2007				
≥ 2008	0.54 (0.44–0.66)	< 0.001	0.60 (0.48–0.75)	< 0.001

*Note:* The models in part A of the table represent the analyses without random imputation of missing values. The models in part B of the table represent the analyses with random imputation of missing data.

^a^ Univariate and multivariate logistic regression.

### 3.1 Machine Learning

The variable importance was estimated using a Random Forest model, shown in Figure 1B. The most important variable was found to be the occurrence of loco‐regional recurrences. Moreover, the machine learning experiment demonstrated that small signals could be recognized, as seen in Table 6, further enforcing what is already found with the predictive factors analysis. The logistic algorithm performed best, providing a slight advantage, though its clinical significance remains limited. However, this can be considered as a baseline for further developments where the information collected from additional sources, like digital slides, will be exploited for relapse prediction alone or in a multimodal modality together with clinical data.

**TABLE 6:** Performance comparison of machine learning algorithms for detecting small signals in predictive factors analysis.

Algorithm	Accuracy	*K*	*F*‐measure	AUROC
NaiveBayes	54.85%	0.0917	0.543	0.584
SVM	54.26%	0.0685	0.481	0.534
Logistic	55.32%	0.1035	0.552	0.584
Multilayer Perceptron	54.02%	0.0803	0.540	0.556
K*	54.26%	0.0840	0.542	0.541
AdaBoost	53.19%	0.0605	0.530	0.565
Bagging	54.49%	0.0870	0.543	0.560
RandomForest	52.84%	0.0523	0.525	0.558

*Note:* This table presents the accuracy, kappa statistic (*K*), *F*‐measure, and area under the receiver operating characteristic curve (AUROC) for various algorithms, including NaiveBayes, SVM, Logistic Regression, Multilayer Perceptron, K*, AdaBoost, Bagging, and Random Forest. The Logistic Regression algorithm shows a slight advantage in performance, though not reaching clinical significance.

### 3.2 Correlations Between Local Recurrences and Different Distant Metastasis Locations

Figure 1C illustrates the network of correlations between loco‐regional recurrences and distant metastases in bone, liver, lung, brain, and other sites. Loco‐regional recurrences have significant positive correlations with the most metastatic sites (*p* < 0.05) but brain metastases, which do not show any significant correlation. There were significant positive correlations between bone, lung, liver, and brain metastases (*p* < 0.05) but negative correlations with other metastatic sites (*p* < 0.05).

## 4 Discussion

### 4.1 Key Results

Key findings revealed that factors such as lobular invasive histotype, triple‐negative molecular subtype, higher tumor grade, larger tumor size, lymph node involvement at diagnosis, and the occurrence of loco‐regional recurrences represent significant risk factors for further metastasis development. Conversely, the expression of progesterone receptors was found to be protective. Machine learning analyses supported these findings, highlighting the occurrence of loco‐regional recurrences as a critical predictor for metastasis, although the models' clinical significance was limited. Additionally, significant correlations were observed between loco‐regional recurrences and most metastatic sites, excluding brain metastases. Moreover, patients who developed distant metastases had significantly worse overall survival (23.62% at 20 years) compared to controls (99.23%, *p* < 0.01).

### 4.2 Interpretation and Comparison With the Literature

In alignment with our findings, Min et al. found lobular invasive carcinoma histotype to be a significant risk factor for metastatization, independent from loco‐regional lymph node involvement, molecular subtype, and other factors [29]. In contrast to Min et al. and Anwar et al. [29, 30], but in accordance with Xiao et al. and Onitilo et al. [31, 32], we found the triple‐negative breast cancer molecular subtype to be a risk factor for distant metastasis in both univariate and multivariate analysis. Actually, Min et al. found it to be significant only in the univariate analysis [29]. According to Min et al. (2021) and Park et al. (2014), after imputation of the missing values, we found the luminal B HER2+ molecular subtype to be a significant predictive factor for distant metastasis in the multivariate analysis [29, 33]. Furthermore, as other authors did in previous literature, we confirmed as predictive factors for distant metastases development larger tumor size and lymph node involvement at diagnosis [29, 30, 31, 33, 34, 35], as well as higher tumor grade [29, 31]. However, high tumor grade was not always found to be an independent risk factor for distant metastasis [30, 33, 34, 35]. According to previous literature, we found a significant role of loco‐regional recurrences in the prediction of distant ones [33, 34, 36, 37, 38].

According to Purdie et al., progesterone receptor expression served as a significant protective factor in breast cancer, particularly in early‐stage disease [39]. The study of Purdie et al. demonstrated progesterone receptors to be associated with improved overall and disease‐free survival [39]. This protective role is evident among estrogen receptor‐positive breast cancer patients, where PR positivity further stratifies patients into those with a more favorable prognosis [39]. The same finding was also confirmed by other authors, corroborating our result that showed a significant predictive value of progesterone receptor expression in reducing the risk of distant metastatization [31, 39, 40, 41, 42].

Treatment strategies employed for breast cancer, including surgery and chemotherapy, are highly correlated with the underlying tumor and patient characteristics, as these factors drive the clinical decision‐making process. Our data showed that patients who developed distant metastases during their follow‐up were more likely to have undergone radical surgical interventions and adjuvant chemotherapy. These patients typically presented with larger tumors, with massive lymph node involvement at diagnosis (pN3), higher proliferation indexes (Mib1/Ki‐67 > 20%), higher prevalence of high grading (G3), and aggressive subtypes such as luminal B and triple‐negative ones. The multivariate analysis further confirmed that these basal tumor characteristics were significant predictive factors for the development of metastases.

Machine learning has shown in previous literature various utilizations ranging from identification of metastatic cancers using unstructured electronic medical notes or structured databases, as well as to predict the development of distant metastases [43, 44, 45, 46, 47, 48, 49, 50]. Logistic regression models were among the most commonly used models and, in our analysis, they resulted in the most accurate. However, other authors found other models, such as Random Forest or XGBoost algorithm, to be the most predictive. Duan et al. highlighted the use of the Random Forest algorithm to analyze gene expression data, offering high predictive accuracy and supporting personalized interventions [43]. Similarly, Lu et al. emphasized the value of the XGBoost algorithm in integrating clinical and radiomic data, leading to precise risk assessments that improve treatment strategies [44]. Soliman et al. further illustrated how machine learning, combined with artificial intelligence in pathology, can identify complex patterns in clinical, pathological, and imaging data, facilitating more targeted interventions [45]. Deep learning might also be exploited to identify breast cancer features usually assessed via immunohistochemistry but directly from morphology, like for example HER2 [51]. Moreover, Wang et al. demonstrated the efficacy of deep learning models in analyzing ultrasound features to accurately identify patients at risk for metastasis, thus supporting personalized treatment planning [46]. Zhong et al. also underscored the role of machine learning, particularly through XGBoost, in analyzing clinical and pathological data to predict both diagnosis and survival outcomes in patients with bone metastasis [47]. Additionally, Ling et al. (2019) showed how natural language processing (NLP) combined with machine learning can extract valuable insights from electronic medical records, improving the accuracy of identifying metastatic cases and enabling better risk stratification [48]. Furthermore, Nicolò et al. highlighted the integration of machine learning with mechanistic modeling, enhancing relapse and metastatic progression predictions, thereby optimizing personalized treatment strategies [49].

Collectively, these studies underscored the extremely transformative potential of machine learning in improving prognostic assessments and guiding tailored treatment approaches in breast cancer care. In many instances, the predictivity of the presented machine learning models was higher than physicians' accuracy. However, some authors included in the models not only the basal information but also the treatment information (e.g., radiotherapy, chemotherapy) [47]. As previously seen, tumor management is highly correlated with tumor basal information such as tumor subtype, grading, etc. In our study, we excluded tumor treatments from the predictive models to avoid possible multi‐collinearity and overfitting. In other studies, specific types of information not usually available in common practice were used, such as expression arrays, radionics, and additional immunohistochemical markers [43, 44, 46, 49]. These results led us to assume that incorporating additional information from standard pathological findings or standard imaging diagnostics could further support the routine data we used in the prediction effort, thereby improving the performance of the tested models.

The network of correlations between local recurrences and distant metastasis sites revealed significant relationships between loco‐regional recurrences and various distant metastatic sites, except for brain metastases, which appear to follow a distinct correlation pattern. The higher prevalence of loco‐regional recurrences in patients who developed distant metastases during their follow‐up again underscores the need for careful monitoring. This evidence suggests that tumors with greater biological aggressiveness, or those diagnosed at more advanced stages, may have an intrinsic tendency for loco‐regional recurrence, which could indicate a higher risk for distant spread. However, the hematogenous and lymphatic pathways of disease dissemination are not always correlated and may operate independently, indicating the complexity of metastatic patterns [4]. Moreover, our findings align with the results of Hicks et al., who also reported a significant positive correlation between lung and brain metastases [52].

### 4.3 Strengths and Weaknesses

The strengths of our study include the sample size, its homogeneity, and the significant follow‐up length. The patients were all treated by the same specialists working at a reference breast unit since the early 2000s. The study is limited by the variation in treatments over the past two decades, especially non‐surgical approaches, due to the substantial advancements in cytotoxic and biological therapies in both adjuvant and neoadjuvant contexts. Despite improvements in survival metrics, previous studies indicate that metastatic site patterns have not significantly altered over time [53]. The evidence suggests that treatment effects likely exerted only a limited influence on our findings. However, the retrospective design of this study limits our access to comprehensive information about chemotherapy management. Notably, employing the time period as a proxy for treatment evolution and adjusting for it in the multivariate analysis did not change the significance of the identified risk factors. Another limitation, for the same reason, is the sample size concerning certain specific information, such as genetic testing. In fact, due to the increasingly systematic performance of genetic testing to detect mutations in genes that most commonly predispose to breast cancer (e.g., BRCA1, BRCA2, PTEN, P53, etc.), only 60 patients treated from 2001 to 2015 underwent such testing, simply because it was not widely applied during that period of time, mainly for cost–benefit reasons. Consequently, the low number of patients with genetic testing prevents us from drawing robust conclusions regarding mutated women and the associated hereditary risk factors.

### 4.4 Generalisability, Clinical Relevance, and Directions for Future Research

The generalisability of our findings is supported by the identification of key predictive factors for distant metastasis in breast cancer patients, including the triple‐negative molecular subtype, G3 grading, larger tumor size, lymph node involvement at diagnosis, and loco‐regional recurrence. These factors commonly indicate a more aggressive tumor biology and are likely applicable across diverse patient populations. Clinically, this research underscores the importance of tailoring follow‐up strategies to earlier detect metastases, especially in patients at higher risk due to these aggressive features. Recognizing distinct patterns of metastatization, such as the unique spread of brain metastases, can further refine surveillance and treatment protocols, particularly for oligometastatic patients who may benefit from targeted surgical interventions or advanced systemic therapies. However, it should be noted that the limited genetic testing data restricts the generalisability of our conclusions to mutated women, necessitating further studies to clarify hereditary risk factors in this subgroup.

Future research should, prospectively, focus on clinical management and the timing and specific locations of distant metastasis, considering the significant interrelationships identified among metastatic sites, including bones, liver, and lungs. A more precise understanding of these patterns may facilitate the development of personalized follow‐up and treatment plans. Our findings reveal a distinct relationship between loco‐regional and distant recurrence, suggesting that the biological mechanisms underlying local and distant metastatic progression may involve multiple factors. A recent study indicates that complete axillary dissection, in comparison to sentinel lymph node biopsy, may negatively impact survival in patients receiving neoadjuvant therapy [54]. This finding supports the hypothesis that less invasive axillary surgery may reduce systemic inflammatory responses or better preserve immune function, potentially mitigating the risk of distant metastases. Furthermore, our results likely reflect the intricate relationship between tumor biology and the host immune response. Nonetheless, the retrospective design of our study complicates the separation of surgical treatment effects from tumor biology, as these factors are closely interconnected, which poses challenges for statistical analysis and the interpretation of their respective contributions.

Additionally, exploring the potential of artificial intelligence in predicting metastasis risk based on histopathological imaging, radiomics, clinical, and pathological data using standard available information could revolutionize individualized patient care, offering more accurate risk assessments and tailored therapeutic approaches, in order to definitely enhance early disease detection and optimize treatment outcomes in breast cancer management. Pre‐trained machine learning models for predicting distant metastases in breast cancer provide a solid foundation; however, they necessitate further enhancement through the incorporation of various data sources, including radiological and pathological imaging, among others. While traditional logistic regression models offer static risk assessments, machine learning‐based models are dynamic and can improve over time when integrated into a clinical management system. Embedding these models through platforms like Shiny applications makes real‐time risk assessment feasible. Moreover, establishing a robust monitoring framework to track key performance metrics and trigger timely retraining is essential to maintain and enhance the models' clinical utility and relevance.

## 5 Conclusions

Our findings revealed several significant risk factors for distant metastasis development, including lobular invasive histotype, triple‐negative molecular subtype, lobular B HER2+ subtype, high tumor grade, larger tumor size, lymph node involvement at diagnosis, and loco‐regional recurrence. These factors likely indicate a more aggressive tumor behavior at diagnosis, which contributes to a higher risk of distant recurrence during the follow‐up. Furthermore, the expression of progesterone receptors was found to be a significant protective factor against metastasis formation. Our study also looked into the relationship between loco‐regional recurrences and distant ones and found that loco‐regional recurrences were significantly correlated with most metastatic sites, except brain metastases, which appeared to follow their own distinct pattern. Additionally, the application of machine learning models revealed essential insights into the significance of these factors, highlighting loco‐regional recurrences as a critical predictor of metastasis. While the logistic algorithm demonstrated the best performance among the tested models, the machine learning approach overall provides a promising foundation for future development, potentially incorporating additional data sources to improve the accuracy and clinical utility of metastasis prediction in breast cancer patients.

## Author Contributions

Substantial contributions to conception and design, or acquisition of data, or to analysis and interpretation of data (S.B., A.P.L., G.V., V.D.M.). Drafting the article, or revising it critically for important intellectual content (M.O., L.M., C.C., E.P., C.D.L.). All authors have read and approved the final manuscript.

## Ethics Statement

The present study (037/2019) was approved by the internal review board of the Department of Medical Area (University of Udine). It was conducted per the Helsinki Declaration and followed the dictates of the general authorization to process personal data for scientific research purposes by the Italian Data Protection Authority.

## Consent

According to national legislation, the need for informed consent was waived by the IRB listed above because this was a retrospective cohort study.

## Conflicts of Interest

The authors declare no conflicts of interest.

## Data Availability

The data that support this study's findings are available. However, restrictions apply to the availability of these data, which were used under license for the current study and are not publicly available. Data are, however, available from the authors upon reasonable request and with permission of the Internal Review Board.
